# Understanding the psychosocial impact of weight loss following bariatric surgery: a qualitative study

**DOI:** 10.1186/s40608-018-0215-3

**Published:** 2018-12-03

**Authors:** Dina H Griauzde, Andrew M Ibrahim, Natalie Fisher, Amanda Stricklen, Rachel Ross, Amir A Ghaferi

**Affiliations:** 1 Ann Arbor VA Health System, Ann Arbor, MI USA; 2 Department of Internal Medicine, University of Michigan, 2800 Plymouth Road, Building 16, Room 16-278C, Ann Arbor, MI 48109-2800 USA; 3 Department of Surgery, University of Michigan, Ann Arbor, MI USA; 4 Institute for Healthcare Policy & Innovation, University of Michigan, Ann Arbor, MI USA

**Keywords:** Obesity, Weight loss surgery, Psychosocial

## Abstract

**Background:**

Bariatric surgery leads to changes in mental health, quality of life and social functioning, yet these outcomes differ among individuals. In this study, we explore patients’ psychosocial experiences following bariatric surgery and elucidate the individual-level factors that may drive variation in psychosocial outcomes.

**Methods:**

Eleven semi-structured focus groups with Michigan Bariatric Surgery Collaborative (MBSC) patients (*n* = 77). Interviews were audio recorded, transcribed verbatim, and analyzed using a grounded theory approach. Data on participant demographic characteristics were abstracted from the MBSC clinical registry.

**Results:**

Most focus group participants were female (89%), white (64%), and married (65%). We identified three major themes: (1) change in self-perception; (2) change in perception by others; and (3) change in relationships. Each theme includes 3 sub-themes, demonstrating a range of positive and negative psychosocial experiences. For example, weight loss led to increased self-confidence among many participants while others described a loss of self-identity. Some noted improved relationships with family or friends while others experienced worsening or even loss of relationships due to perceived jealousy.

**Conclusion:**

Weight loss following bariatric surgery leads to complex changes in self-perception and inter-personal relationships, which may be proximal mediators of commonly assessed mental health outcomes such as depression. Individuals considering bariatric surgery may benefit from anticipatory guidance about these diverse experiences, and post-surgical longitudinal monitoring should include evaluation for adverse psychosocial events.

## Background

The public health burden of obesity is undeniable [1]. Within the United States, over one-third of adults live with obesity [2] and worldwide over six hundred million adults – 11% of men and 15% of women – live with obesity [3]. Further, a growing number of individuals live with morbid or severe obesity (body mass index > 40 kg/m^2^ [4, 5]), which leads to serious health consequences and contributes significantly to obesity-related health care spending [6]. Bariatric surgery, which includes gastric bypass, sleeve gastrectomy, and laparoscopic gastric banding, is the most effective treatment for morbid obesity [7, 8], often reducing excess weight within one year of surgery for a prolonged duration [9]. Post-surgical weight loss predictably leads to resolution of and/or improved control over obesity-related chronic conditions such as type 2 diabetes mellitus [10, 11].

In addition to poor physical health, individuals with obesity are also more likely to experience poor mental health [12–14] and social dysfunction secondary to factors such as weight-related stigmatization, discrimination, and poor body image [15–17]. Psychosocial changes also occur following major weight loss after bariatric surgery [18]. However, in contrast to the predictable physical health benefits of major weight loss, psychosocial outcomes differ among patients and little is known about the individual-level experiences that may contribute to these variations. For example, many individuals experience decreased depression severity and increased quality of life following bariatric surgery [19–21]. Yet others may experience psychological benefit only in the near-term [22] or not at all [23]. Some may experience worsened mental health following weight loss surgery with several recent reports demonstrating increased rates of post-surgical self-harm, hospitalization for depression, and binge drinking behavior [24–26].

Poor mental health can compromise post-surgical weight loss [27], and psychosocial functioning is commonly assessed prior to bariatric surgery using standardized tools and a clinical interviews [28]. However, there are no definitive guidelines for this process [29] and the accuracy of these assessments may be undermined by patients’ underreporting of psychological dysfunction [30]. Following bariatric surgery, psychosocial functioning is not routinely assessed in the clinical setting. Among researchers, quantitative psychometric tools are commonly used to assess post-surgical depression severity and health-related quality of life (e.g Impact of Weight on Quality of Life, Short Form Survey 36). While these instruments can elucidate general trends and associations, they fail to capture the lived individual-level experiences that may drive variation in outcomes (e.g. increased marital discord following major weight loss [31, 32]). Hypothesized mechanisms for improved mental health include increased self-confidence, improved self-image, and better relationships as a result of major weight loss [33, 34]. Conversely, worsened mental health may be due to unrealistic pre-surgical expectations about the potential physical and mental health benefits of weight loss [35] or unanticipated changes in social relationships [36].

In this study, we conducted semi-structured focus groups with 77 US adults who underwent bariatric surgery, and we characterize the patient-level experiences that may drive differences in psychosocial outcomes after major weight loss. These data may facilitate more personalized counseling prior to bariatric surgery, and may provide patients with a more nuanced understanding of the potential psychosocial risks and benefits of bariatric surgery within the context of their individual lives. Further, these data may inform post-operative strategies to discuss, identify, and treat psychological dysfunction following weight loss surgery.

## Methods

### Study design

We conducted a qualitative study using semi-structured focus group interviews with patients undergoing bariatric surgery across the state of Michigan. The purpose of these focus groups was to better understand patient experiences after bariatric surgery. Focus group participants were asked to discuss changes in their lifestyle, health, psychological wellbeing, and social relationships. For this manuscript, we focused specifically on the psychological and social changes. This study was approved by the University of Michigan’s Institutional Review Board.

### Participants and recruitment

Patients undergoing bariatric surgery were recruited from four hospitals affiliated with the Michigan Bariatric Surgery Collaborative (MBSC). The MBSC is a statewide clinical registry and quality improvement program in the state of Michigan, which includes more than 70,000 bariatric surgery patients. To ensure a broad range of perspectives, we purposively sampled participants based on gender and geography. MBSC site coordinators identified post-surgical patients who were then contacted by the study team by phone and email. Those who agreed to participate in the focus groups provided written informed consent and received financial compensation for their time.

### Data collection

Eleven focus groups were conducted between February 7, 2014 and March 28, 2014. A professionally trained moderator conducted all focus groups, which each lasted approximately one hour in duration. The semi-structured interview guide was developed in collaboration with patients, bariatric surgeons, medical weight loss specialists, nurses, dieticians, professional organizations (i.e., American Society for Metabolic and Bariatric Surgery and the Society of American Gastrointestinal and Endoscopic Surgeons), and members of a private insurance company. A summary of the focus group questions that pertained to psychosocial experiences are shown in Appendix 1.

Data were abstracted from the MBSC clinical registry for each focus group participant including age, gender, race, date of operation, procedure type (e.g. adjustable gastric band, sleeve gastrectomy, and Roux-en-y gastric bypass, or duodenal switch), marital status, education level, employment status, approximate annual income and estimate weight loss.

### Data analysis

All focus groups were audio and video recorded and transcribed verbatim. Four members of the research team, including a bariatric surgeon (AG), a primary care physician (DG), a surgical resident (AI), and non-physician with a Master’s in Public Health (NF), independently reviewed a subset of transcripts.

**Fig. 1:**
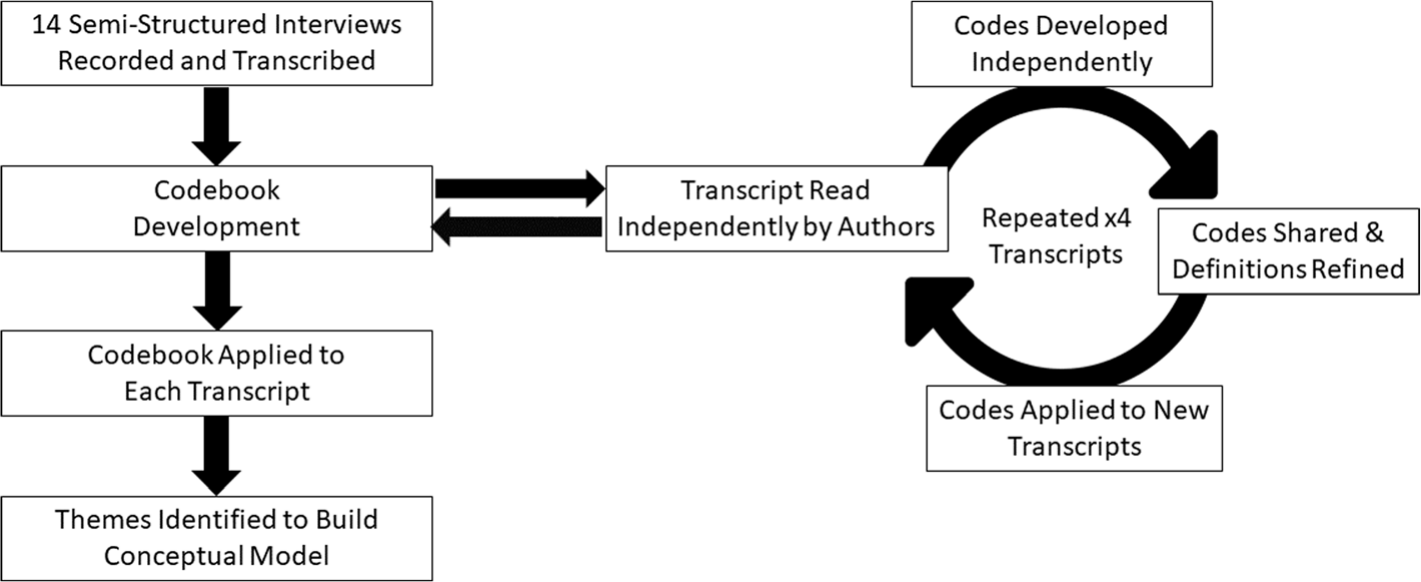
Coding of Transcripts. Each interview was transcribed. A codebook was developed with 4 authors independently reading a transcript, developing codes, then refining code definitions by consensus. After initial developed, codebook as applied to a new transcript, and again refined by author consensus. After 4 iterations of codebook refinement, the remained of transcripts was coded

Codes and definitions were generated during consensus conferences using a grounded theory approach. [37] Specifically, initial codes were created to reflect the main topics in the interview guide, and these were then grouped into themes and sub-themes to reflect the patterns and concepts that emerged from the data. Once the coding scheme was established, all four investigators independently coded each transcript. These investigators then met to review their coding and resolve all differences. Few new codes emerged after reviewing 4 transcripts and no new codes emerged after reviewing 8 transcripts. After 11 focus groups, we reached data saturation, and therefore did not conduct additional focus groups (Fig. 1). We counted the occurrence of each theme and sub-theme to understand their relative frequencies within our data (Appendix 2)*.* All transcripts were coded in NVivo (QSR International), a qualitative data analysis computer software package.

Lastly, we discussed our themes and subthemes by telephone with subset of six MBSC Patient Advisors to ensure that our findings did not overlook important experiences among bariatric surgery patients. The MBSC Patient Advisory Program is a partnership between the MBSC and post-bariatric surgery patients that aims to bring the patient voice to design, operations, and advancements of the MBSC program. The Patient Advisors believed that our findings accurately represented the psychosocial experiences of individuals after bariatric surgery.

## Results

A total of 77 individuals participated in one of 11 focus groups. The mean age of all participants was 48.8 years (range 26 to 72). Most (*n* = 69; 89%) were female, white (*n* = 50; 65%), and married (*n* = 49; 64%). Additional participant characteristics including their post-surgical weight loss and psychiatric conditions are shown in Table 1.

**Table 1:** Characteristics of Focus Group Participants

	No.	Percent (Column)
Total Participants	77	100.0%
Female	69	89.6%
Age (Mean)	48.8	–
Race		
White	50	64.9%
African American	16	20.8%
Other / Not-Reported	11	14.3%
Procedure Type		
Sleeve Gastrectomy	25	32.5%
Gastric bypass	22	28.6%
Lap-Band	20	26.0%
Duodenal Switch	10	13.0%
Time Since Surgery		
Less than 1 Year	20	26.0%
1 to 2 Years	9	11.7%
2 to 3 Years	12	15.6%
3 to 4 Years	12	15.6%
4 to 5 Years	6	7.8%
More than 5 years	15	19.5%
Marital Status		
Married	49	63.6%
Widowed	8	10.4%
Divorced	7	9.1%
Single	2	2.6%
Education Level		
High School Graduate	8	10.4%
Some College	30	39.0%
College Graduate	14	18.2%
Graduate Degree	13	16.9%
Employment Status		
Working	50	64.9%
Retired	6	7.8%
Disabled	5	6.5%
In-School	2	2.6%
Homemaker	2	2.6%
Income		
Less than $25,000	7	9.1%
$25,000 to $49,999	22	28.6%
$50,000 to $75,000	12	15.6%
More than $75,000	24	31.2%
Any Psychiatric Diagnosis *	35	49.3%
Bi-polar diagnosis	1	1.4%
Anxiety diagnosis	11	15.5%
Depression diagnosis	32	45.1%
Substance Abuse	2	2.8%
Eating Disorder	0	0.0%
Other psych diagnosis	0	0.0%
Weight Loss**		
	Mean	Std. Dev.
1 Year Absolute wt. loss, lbs.	91.0	37.2
1 year % wt. lost	32.0	10.4
1 year % excess wt. lost	63.9	21.8

*Data on psychologic diagnoses at the time of surgery was available for 71 of our patients. **1-year follow data regarding weight loss was available for 52 of the patients who participated in our study

Within our data, we identified 3 major themes: (1) change in self-perception (2) change in perception by others and (3) change in relationships. Within each major theme we identified three subthemes, which are described below and illustrative quotes for each are shown on Table 2. Each theme was present across each of the procedure types after the patient experience weight loss.

**Table 2:** Representative Quotations from Major Themes and Sub-Themes

Theme 1**:** Change in Self-Perception	
*“When I Look in the Mirror”*	
“I don’t feel like a different person. I don’t see it most of the time. I can put on one of my shirts that I could hardly button before I had surgery…it’s the only time I can see [the weight loss]...now it’s like a dress.”	
“I went in to try to find a pair of jeans because people were complaining that my butt was so saggy. And I said I wear a size 17 and they’re like, no you don’t. I came out in a 9–10, and I still can’t believe that.”	
*“I Have More Confidence”*	
“I would say that my friendships have gotten better. [My friends,] they loved me no matter what, and I just think my confidence has grown so much so that I have opened up to them and have a better connection with them now.”	
“I would’ve considered myself a wallflower before because I was embarrassed with how I looked. And I would imagine what people would think looking at me, you know. No more, no more. About a year and a half ago, I went to a wedding, and I’m dancing. I’m dancing fast. I’m dancing, I’m smiling the whole time. I danced the whole night, I made it the whole night dancing.”	
*“I Used Food to Cope”*	
“I’m losing the weight…I’m going back to the old me because I understand what happened throughout my life to make me emotionally eat and drink to gain weight. I’m embracing the change, mentally being healthier and more stable, knowing why I became overweight.”	
“[Before surgery] I could just eat a pack of cookies, get sick, whatever, and pass out like an alcoholic. [Now] the surgery limits how much food I can intake. So it made me go to counseling and start dealing with the underlying issues that were causing me to overeat.”	
Theme 2. Change in Perception by Others	
*“You Took The Easy Way Out”*	
“I work in the ER and I told everyone at work I was having the surgery and I was really surprised with some of the backlash that I got. They’re like, ‘You’re not trying hard enough. You need to run. You need to do this. You need to diet.’”	
“I had one friend [that would tell people that I had bariatric surgery.] It was like a flipping dirty word. Finally I said, ‘Why do you have to tell people that? It’s not their business. That’s my business.’ [And she said,] ‘Well, they should know.’”	
*“Are You New Here?”*	
“I have people at work that would see me and say, are you new? And I’m like, no, I’ve been here for like 12 years.”	
“I went home to my dad’s funeral two years ago and the number of people who didn’t know me really upset me...people who have known me all my life walked right by me. Even as I walked right up to them said ‘Hi, I’m Nicole...I’m Nicole. You’ve known me since I was a babe in arms.’”	
*“I’m no longer invisible”*	
“I’m being perceived in a more professional way. I can dress differently now and I’m finding that I’m trying to take on new projects. I feel that professionally, I might have more opportunities for advancement just because I look different. Sad, but true. It’s the way of the world.”	
“People would let a door slam in your face before. I would sit on bleachers at my kids’ events and people didn’t look at me, didn’t talk to me, nothing. Now they’re like, ‘Hey, let’s go for dinner after this.’”	
Theme 3: Change in Relationships	
*“It made my relationship stronger”*	
“My marriage is great. He is going to love me no matter what but I feel better in my marriage because I feel healthier.”	
“My husband and I have been together since we were 16 years old so he basically watched me go down and come back up…When we’re intimate now I’m not afraid to take my shirt off. We went years with the lights off and the clothes were on as much as possible and that kind of thing.”	
*“They Didn’t Like the Change”*	
“My engagement ended, because he didn’t like the weight loss. At first it was, ‘Have the surgery’ and then he didn’t like how I looked after the weight loss.”	
“I wish somebody would’ve told me how it was going to change my marriage, how it was going to change all my relationships that existed, and the ones that didn’t even exist. It changed my marriage. I almost got a divorce because I was changing so quickly.”	
*“They’re Jealous of Me”*	
“They don’t verbally say they’re jealous but you can kind of tell that they are getting jealous because you’re getting more attention now than they normally do. People treat you a lot differently. They open doors for you. They say hi to you.”	
“[My friend is] seeing a guy and she was like, ‘I don’t want him to meet you because he’s going to fall in love with you’ .... We’ve been friends for a really long time, but that was my first inkling of a little bit of a change in the relationships, which was kind of weird for me.”	

### Change in self-perception

Participants described changes in how they saw themselves after bariatric surgery.

#### “When I Look in the Mirror”

Many patients reported discordance between their objective post-surgical weight and their perceived body image and identity. One patient stated, “I think for me the mind body experience is so separate. The body loses weight but the mind still stays [the same]” and another endorsed a sense of lost self-identity, stating “once I lost over 100 pounds, and looked in the mirror at myself, I didn’t know that person looking back, and that frightened me.” In several instances, participants described persistent dissatisfaction with their body image despite marked weight loss and comments from others that “you look so good.”

#### “I Have More Confidence”

Many individuals described increased self-confidence after weight loss, which resulted in an increased ability to engage professionally and socially. One participant noted, “I had more confidence to get a better job” and another described a general willingness to embrace new opportunities: “[Now] I don’t have any limits; I’m willing to try whatever.” Several individuals reported improved mental health, which one woman who struggled with depression for 25 years attributed to “just being more confident.”

#### “I Used Food to Cope”

Some participants expressed insight into the psychological factors that contributed to their pre-surgical weight. Several individuals recognized that they used food to cope with emotional distress, and after surgery, “you have to deal with the real reasons you overeat and why you mistreat your body.” Some participants sought professional counseling after bariatric surgery to develop new strategies to manage negative emotions, and one individual indicated that she worked with a therapist prior to surgery to prepare for this change.

### Change in perception by others

Participants also described changes in how they interacted with and were perceived by other individuals after bariatric surgery.

#### “You Took The Easy Way Out”

Some participants felt that they were negatively judged for their decision to have weight loss surgery by friends, family, or co-workers. Bariatric surgery was considered to be the “easy way out” and participants were frequently told “you’re not trying hard enough [to lose weight].”

#### “Are You New Here?”

Many noted that they were not recognized by acquaintances after weight loss. This prompted various emotional responses among focus group participants. While some enjoyed the “actual shock reactions of people” some were saddened by the lack of recognition. One woman said, “I was really upset people who have known me all my life walked right by me.”

#### “I Am No Longer Invisible”

Participants experienced positive changes in how they were treated both professionally and socially, which often led to mixed sentiments. As one participant stated, “I’ve got this new life and it’s exciting, but then you also see how unfair society can be. You’re treated considerably different…people that wouldn’t even open a door for you previously are now opening doors.” Another commented that “[weight loss] is like winning the lottery; people who used to ignore you now want to be your best friend.”

### Change in relationships

Patients in the focus groups discussed several important changes in their relationships following bariatric surgery.

#### “It made my relationship stronger”

Some noted that certain relationships strengthened following weight loss, which was often due to increased self-confidence. In other instances, focus group participants attributed this improvement to a significant other’s concomitant weight loss and their ability to partake in new activities together. One participant commented that “[my husband] lost 60 pounds and we’ve both just turned our whole lifestyle around.”

#### “They Didn’t Like the Change”

In contrast, many participants experienced distress in certain relationships following weight loss. This was often attributed to changes in social activities, which were previously centered around eating. Others indicated that their loved ones preferred their pre-surgical weight. For example, one participant recalled her husband stating, “she can’t get too small, doc, because that’s why I married her. I wanted her that size” and another said “I almost got divorced because I was changing so fast.”

#### “They’re Jealous of Me”

Finally, many participants experienced jealousy from friends and family due to weight loss. For example, one told her younger brother, “Look, I lost 39 pounds but don’t tell mom or sis because they’ll get upset because I’m dropping it so fast.” Others reported receiving increased positive social attention, which resulted in jealousy from friends.

## Discussion

Through focus groups with patients who underwent bariatric surgery, we characterized the complex changes in psychological and social functioning that occur following major weight loss. Participants described changes related to three major themes: (1) self-perception; (2) perception by others; and (3) social relationships. Each of these themes includes positive and negative experiences that were often interrelated and experienced simultaneously by patients. Taken together, the experiences described by our focus group participants may help to explain the differences observed in recent quantitative studies examining the psychosocial impact of weight loss after bariatric surgery.

Several of our focus group participants noted that their depressive symptoms resolved following bariatric surgery. These findings are consistent with prior literature demonstrating a decrease in the prevalence and severity of mental health conditions following bariatric surgery [38, 42] as well as improved mental health and health-related quality of life two years after bariatric surgery [19, 20, 39]. Other participants discussed positive psychosocial changes that may mediate improvements in mental health and quality of life such as increased self-confidence and improved relationships with spouses, friends, co-workers and social acquaintances. Notably, these experiences support previously hypothesized mechanisms for improved mental health following major weight loss [33, 34]. Changes in self-perception described by participants this study also help put into context recent large registry research demonstrated that a large portion of partients aftery bariatric surgery have a change in relationship status (i.e. married patients become divorces or single individuals get married.) [43]

While mental health commonly improves following bariatric surgery [19–21], some individuals never have this benefit [23] and others may experience worsened mental health, including increased risk of self-harm [24–26]. Because these prior studies use administrative data and/or quantitative assessments to measure outcomes, they cannot provide insight into the individual-level factors that may either mitigate or potentiate adverse psychosocial outcomes. Prior qualitative work provides limited insight into the live patient experience, and demonstrates that some individuals are dissatisfied with their body image following surgery [40, 41], which may be due to unrealistic pre-surgical expectations [35]. For others, weight loss following bariatric surgery may negatively alter marital dynamics, even leading to divorce in some circumstances [32]. Our findings substantiate and extend these prior qualitative finds among a large patient cohort. We corroborate findings that relationships with significant others may change following major weight loss [43], and we also provide new insight into changes that may occur in other social and professional settings.

Notably, the psychosocial experiences shared by focus group participants could not be dichotomized as “positive” or “negative” due to the complexity of lived experiences. For example, many were pleased with their objective weight loss, but simultaneously struggled with a loss of self-identity. Others described social and professional gains, but also endorsed resentment for not being treated with similar respect before weight loss. This tension between positive and negative psychosocial experiences may explain, at least in part, why positive psychosocial outcomes diminish over time for some [22]. Future work could examine fluctuations in psychosocial experiences – perhaps in conjunction with weight loss and maintenance – to inform the development of targeted support strategies to optimize physical and mental health outcomes.

Our findings should be interpreted in the context of important limitations. First, similar to other qualitative studies, this manuscript may not be generalizable to the broader population of bariatric surgery patients. To minimize this limitation, we purposefully sampled patients throughout the state of Michigan to capture differences in various characteristics including age, gender, race, and socioeconomic status. The collaborative, while only one state, is amongst the largest collaboratives in the country with a diverse case-mix. Second, it is possible that participants with more favorable surgical outcomes participated in this voluntary study and therefore our findings may under-represent the negative psychosocial impact of bariatric surgery. However, we did reach thematic saturation with regards to negative psychosocial factors. Moreover, we reviewed our findings and conceptual model with a subset of bariatric patients to ensure important themes were not overlooked. Third, participants’ responses could have been influenced by recall or social desirability biases. We aimed to limit this bias by utilizing a trained moderator that had no association with the clinical providers within the MBSC. Finally, we did not assess patients at multiple intervals post-operatively, and their response to weight loss may have differed over time. To minimize this potential time-exposure bias, we made sure our sample included patients at multiple points along the post-operative time line. In that context, we did not notice significant thematic differences across different time points after surgery.

Our study has important implications for patients, providers, policymakers. Among patients, these findings may facilitate a more nuanced understanding of the psychosocial changes that may follow weight loss surgery. The themes identified in this study may help providers identify adverse psychosocial outcomes that may otherwise go undetected. While standardized instruments (e.g., PHQ-9) may be used to screen for common mental health conditions (e.g. depression) these measures may not reveal other psychosocial experiences (e.g. marital discord or judgment by former friends), which may contribute to general wellbeing and achieved weight loss [12, 23]. To elicit this information, providers may consider asking patients questions such as “How has your weight loss changed your relationships?” or “Has anyone judged you negatively for having weight loss surgery?” Lastly, among payers, there are opportunities to promote favorable psychosocial outcomes through reimbursement for post-surgical mental healthcare, which may include support groups, couple’s therapy, or one-on-one counseling.

## Conclusions

Our study provides new insight into the psychosocial experience of patients following bariatric surgery. By understanding these lived experiences, providers may be better equipped to ascertain and address factors that may contribute to psychosocial dysfunction. Prior to surgery, patients and providers may engage in a more personalized discussion of potential psychosocial risks and benefits of surgery, and anticipatory guidance may be better tailored to the patient’s unique circumstances. For example, a patient whose social life revolves around dining out may be encouraged to explore alternative activities *before* surgery and to pre-emptively discuss this lifestyle change with friends and family. Post-operatively, bariatric surgery teams and primary care providers may more effectively elicit and address psychosocial struggles by discussing patients’ changes in self-perception and relationships with others. As rates of severe obesity and surgical treatment for the condition continue to rise, these steps are critical to optimize psychosocial functioning among patients who consider and undergo weight loss surgery.

## Appendix 1

**Table 3:** Focus Group Semi-Structured Interview Guide

Life Changes Since Surgery	
• How have things changed for the better in your life and since you had bariatric surgery? What things have changed for the worse?	
• What were some of the surprises? What changes occurred in your life which were unexpected?	
• Just focusing on your health, what effects has bariatric surgery had on your health?	
• How about your social life and relationships, what specific effects did the surgery have in this area?	
• How about any lifestyle changes, your activities, interests, etc., how has the surgery changed this aspect of your life?	
• Thinking about your emotions and personality and the way you feel, how has the surgery affected this part of your life?	
• Probe any other psychological related changes Overall what would you say are some of the things you struggle with the most since your surgery?	

## Appendix 2

**Table 4:** Frequency of Codes Organized by Themes and Subthemes

	Frequency (No.)	Frequency, Within Theme (Column %)	Frequency, Overall (Column %)
Change in Self-Perception	72	100.0%	47.7%
I have more confidence	31	43.1%	20.5%
When I look in the mirror	23	31.9%	15.2%
I used food to cope	10	13.9%	6.6%
Other	8	11.1%	5.3%
Change in Perception by Others	41	100.00%	27.2%
I felt invisible when I was heavy	22	53.66%	14.6%
You took the easy way out	10	24.39%	6.6%
Are you new here?	5	12.20%	3.3%
Other	4	9.76%	2.6%
Relationship Impact	38	100.0%	25.2%
They’re jealous of me	15	39.5%	9.9%
They didn’t like the change	12	31.6%	7.9%
It made my relationship stronger	8	21.1%	5.3%
Other	3	7.9%	2.0%
Total	151		100.0%

Frequency table was used to guide our descriptive language in the manuscript text: > 50% was considered “Most”; 30–49% “Many”; 10–29% “Some”; < 10% “Few”

## Abbreviations

MBSC: Michigan Bariatric Surgery Collaborative

## References

[1] Flegal KM, Kruszon-Moran D, Carroll MD, Fryar CD, Ogden CL (2016). Trends in obesity among adults in the United States, 2005 to 2014. JAMA.

[2] Adult Obesity Facts | Overweight & Obesity | CDC, (2017). https://www.cdc.gov/obesity/data/adult.html (accessed November 27, 2017).

[3] WHO | Obesity and overweight, WHO. (n.d.). http://www.who.int/mediacentre/factsheets/fs311/en/ (accessed November 16, 2017).

[4] Defining Adult Overweight and Obesity | Overweight & Obesity | CDC, (n.d.). https://www.cdc.gov/obesity/adult/defining.html (accessed November 28, 2017).

[5] Sturm R, Hattori A (2013). Morbid obesity rates continue to rise rapidly in the US. Int J Obes.

[6] Andreyeva T, Sturm R, Ringel JS (2004). Moderate and severe obesity have large differences in health care costs. Obes Res.

[7] Buchwald H, Avidor Y, Braunwald E, Jensen MD, Pories W, Fahrbach K, Schoelles K (2004). Bariatric surgery: a systematic review and meta-analysis. JAMA.

[8] Chang S-H, Stoll CRT, Song J, Varela JE, Eagon CJ, Colditz GA (2014). The effectiveness and risks of bariatric surgery: an updated systematic review and meta-analysis, 2003-2012. JAMA Surg.

[9] Chang SH, Stoll CR, Song J, Varela JE, Eagon CJ, Colditz GA (2014). The effectiveness and risks of bariatric surgery: an updated systematic review and meta-analysis, 2003-2012. JAMA surgery.

[10] Morgan DJR, Ho KM, Armstrong J, Litton E (2015). Long-term clinical outcomes and health care utilization after bariatric surgery: a population-based study. Ann Surg.

[11] Schauer PR, Bhatt DL, Kirwan JP, Wolski K, Brethauer SA, Navaneethan SD, Aminian A, Pothier CE, Kim ESH, Nissen SE, Kashyap SR (2014). STAMPEDE investigators, bariatric surgery versus intensive medical therapy for diabetes--3-year outcomes. N Engl J Med.

[12] Luppino FS, de Wit LM, Bouvy PF, Stijnen T, Cuijpers P, Penninx BWJH, Zitman FG (2010). Overweight, obesity, and depression: a systematic review and meta-analysis of longitudinal studies. Arch Gen Psychiatry.

[13] Sánchez-Villegas A, Pimenta AM, Beunza JJ, Guillen-Grima F, Toledo E, Martinez-Gonzalez MA (2010). Childhood and young adult overweight/obesity and incidence of depression in the SUN project. Obes Silver Spring Md.

[14] Kubik JF, Gill RS, Laffin M, Karmali S (2013). The impact of bariatric surgery on psychological health. J Obes.

[15] Puhl RM, Heuer CA (2009). The stigma of obesity: a review and update. Obes. Silver Spring Md..

[16] Puhl R, Brownell KD (2001). Bias, discrimination, and obesity. Obes Res.

[17] Spahlholz J, Baer N, König H-H, Riedel-Heller SG, Luck-Sikorski C (2016). Obesity and discrimination - a systematic review and meta-analysis of observational studies. Obes Rev Off J Int Assoc Study Obes.

[18] Magdaleno R, Chaim EA, Pareja JC, Turato ER (2011). The psychology of bariatric patient: what replaces obesity? A qualitative research with Brazilian women. Obes Surg.

[19] Strain GW, Kolotkin RL, Dakin GF, Gagner M, Inabnet WB, Christos P, Saif T, Crosby R, Pomp A (2014). The effects of weight loss after bariatric surgery on health-related quality of life and depression. Nutr Diabetes.

[20] Karlsson J, Sjöström L, Sullivan M, subjects S o (1998). (SOS)--an intervention study of obesity. Two-year follow-up of health-related quality of life (HRQL) and eating behavior after gastric surgery for severe obesity. Int J Obes Relat Metab Disord J Int Assoc Study Obes.

[21] Driscoll S, Gregory DM, Fardy JM, Twells LK (2016). Long-term health-related quality of life in bariatric surgery patients: a systematic review and meta-analysis. Obes. Silver Spring Md..

[22] Khandalavala BN, Geske J, Nirmalraj M, Koran-Scholl JB, Neumann-Potash L, McBride CL (2015). Predictors of health-related quality of life after bariatric surgery. Obes Surg.

[23] van Hout GCM, Boekestein P, Fortuin FAM, Pelle AJM, van Heck GL (2006). Psychosocial functioning following bariatric surgery. Obes Surg.

[24] Bhatti JA, Nathens AB, Thiruchelvam D, Grantcharov T, Goldstein BI, Redelmeier DA (2016). Self-harm emergencies after bariatric surgery: a population-based cohort study. JAMA Surg..

[25] Lagerros YT, Brandt L, Hedberg J, Sundbom M, Bodén R (2017). Suicide, self-harm, and depression after gastric bypass surgery: a Nationwide cohort study. Ann Surg.

[26] Morgan DJR, Ho KM (2017). Incidence and risk factors for deliberate self-harm, mental illness, and suicide following bariatric surgery: a state-wide population-based linked-data cohort study. Ann Surg.

[27] Herpertz S, Kielmann R, Wolf AM, Hebebrand J, Senf W (2004). Do psychosocial variables predict weight loss or mental health after obesity surgery? A systematic review. Obesity.

[28] Marek RJ, Heinberg LJ, Lavery M, Merrell Rish J, Ashton K (2016). A review of psychological assessment instruments for use in bariatric surgery evaluations. Psychol Assess.

[29] Snyder AG (2009). Psychological assessment of the patient undergoing bariatric surgery. Ochsner J.

[30] Ambwani S, Boeka AG, Brown JD, Byrne TK, Budak AR, Sarwer DB, Fabricatore AN, Morey LC, O’Neil PM (2013). Socially desirable responding by bariatric surgery candidates during psychological assessment. Surg Obes Relat Dis Off J Am Soc Bariatr Surg.

[31] Ferriby M, Pratt KJ, Balk E, Feister K, Noria S, Needleman B (2015). Marriage and weight loss surgery: a narrative review of patient and spousal outcomes. Obes Surg.

[32] Liu RH, Irwin JD (2017). Understanding the post-surgical bariatric experiences of patients two or more years after surgery. Qual Life Res Int J Qual Life Asp Treat Care Rehabil.

[33] Ghaferi AA, Lindsay-Westphal C (2016). Bariatric surgery--more than just an operation. JAMA Surg..

[34] Ghaferi AA, Woodruff M, Arnould J (2016). The behavior and biology behind bariatric surgery outcomes. JAMA Surg..

[35] Homer CV, Tod AM, Thompson AR, Allmark P, Goyder E (2016). Expectations and patients’ experiences of obesity prior to bariatric surgery: a qualitative study. BMJ Open.

[36] Courcoulas A (2017). Who, why, and how? Suicide and harmful behaviors after bariatric surgery. Ann Surg.

[37] Starks H, Trinidad SB (2007). Choose your method: a comparison of phenomenology, discourse analysis, and grounded theory. Qual Health Res.

[38] Dawes AJ, Maggard-Gibbons M, Maher AR, Booth MJ, Miake-Lye I, Beroes JM, Shekelle PG (2016). Mental health conditions among patients seeking and undergoing bariatric surgery: a meta-analysis. JAMA.

[39] Mamplekou E, Komesidou V, Bissias C, Papakonstantinou A, Melissas J (2005). Psychological condition and quality of life in patients with morbid obesity before and after surgical weight loss. Obes Surg.

[40] Magdaleno R, Chaim EA, Pareja JC, Turato ER (2011). The psychology of bariatric patient: what replaces obesity? A qualitative research with Brazilian women. Obes Surg.

[41] Alegría CA, Larsen B (2015). “That’s who I am: a fat person in a thin body”: weight loss, negative self-evaluation, and mitigating strategies following weight loss surgery. Negative self-evaluation following WLS, J Am Assoc Nurse Pract.

[42] Kalarchian MA, King WC, Devlin MJ (2016). Psychiatric disorders and weight change in a prospective study of bariatric surgery patients: a 3-year follow-up. Psychosom Med.

[43] Bruze G, Holmin TE, Peltonen M (2018). Associations of bariatric surgery with changes in interpersonal relationship status: results from 2 Swedish cohort studies. JAMA Surg..

